# Apremilast ameliorates IL-1α-induced dysfunction in epidermal stem cells

**DOI:** 10.18632/aging.203265

**Published:** 2021-08-10

**Authors:** Yuxi Jia, Xiangru Chen, Jing Sun

**Affiliations:** 1 Department of Dermatology, The China-Japan Union Hospital of Jilin University, Changchun, Jilin 130033, China

**Keywords:** Apremilast, ESCs, IL-1α, oxidative stress, inflammation

## Abstract

Background and purpose: Skin tissue is the natural barrier that protects our body, the damage of which can be repaired by the epidermal stem cells (ESCs). However, external factors abolish the self-repair ability of ESCs by inducing oxidative stress and severe inflammation. Apremilast is a small molecular inhibitor of phosphodiesterase 4 that was approved for the treatment of psoriasis. In the present study, the protective property of Apremilast against IL-1α-induced dysfunction on epidermal stem cells, as well as the preliminary mechanism, will be investigated.

Methods: ESCs were isolated from neonatal mice. The expression levels of TNF-α, IL-8, IL-12, MMP-2, and MMP-9 were detected using real-time PCR and ELISA. MitoSOX Red assay was used to determine the level of mitochondrial reactive oxygen species (ROS). Western blot and real-time PCR were utilized to determine the expression levels of IL-1R1, Myd88, and TRAF6. Activation of NF-κB was assessed by measuring the p-NF-κB p65 and luciferase activity. Capacities of ESCs were evaluated by measuring the gene expressions of integrin β1 and Krt19 using real-time PCR.

Results: Firstly, the expression levels of TNF-α, IL-8, IL-12, MMP-2, MMP-9 and IL-1R1, as well as the ROS level, were significantly elevated by IL-1α but greatly suppressed by treatment with Apremilast. Subsequently, we found that the activated Myd88/TRAF6/NF-κB signaling pathway induced by stimulation with IL-1α was significantly inhibited by the introduction of Apremilast. As a result, Apremilast protected ESCs against IL-1α-induced impairment in capacities of ESCs, this was verified by the elevated expression levels of integrin β1 and Krt19.

Conclusions: Apremilast might ameliorate IL-1α-induced dysfunction in ESCs by mitigating oxidative stress and inflammation through inhibiting the activation of the Myd88/TRAF6/NF-κB signaling pathway.

## INTRODUCTION

As the natural barrier, skin tissue plays an important role in preventing water loss, maintaining a constant body temperature, resisting infection from microorganisms, reducing mechanical injury, providing a protective shield, and maintaining the homeostasis of the body [1, 2]. Skin mainly consists of the epidermis, dermis, subcutaneous tissues, and their accessory organs [3]. Epidermal stem cells (ESCs) are specific stem cells derived from the embryonic ectoderm, which can be differentiated into various layers of the skin to activate the state of proliferation and differentiation and the apoptotic state of the epidermis. In this way, the integrity and dynamic equilibrium of the epidermis is maintained. In addition, as the specific stem cells for wound repairing, ESCs play a vital role in the repairing and rebuilding of skin and its accessory organs [4, 5]. However, ESCs are easily damaged by external stimuli, such as ultraviolet (UV), which induces DNA damage and oxidative stress in ESCs [6]. Interleukin-1α (IL-1α) has been identified as an important DNA damage sensor that transmits the genotoxic stress signal to oxidative stress and inflammation, making IL-1α a potential target for the alleviation of injury on ESCs by UV [7]. As an inflammatory factor, IL-1α induces the excessive production of reactive oxygen species (ROS) [8], cyclooxygenase-2 (COX-2) [9], and prostaglandin 2 (PGE_2_) [10], which are important elements that contribute to the activation of oxidative stress [11, 12]. By mediating the IL-1α/IL-1R signaling pathway, the excretion of inflammatory factors is significantly induced, further triggering the development of multiple inflammation-related diseases [13]. Tracy reported that as a major epithelial alarmin released from lung epithelial cells, IL-1α induces the excretion of inflammatory mediators in fibroblasts during photodynamic therapy [14]. Clinically, the expression of IL-1α is reported to be greatly elevated in the lung tissues of chronic obstructive pulmonary disease (COPD) patients [15]. The level of pro-inflammatory factors in fibroblasts isolated from COPD patients is significantly higher than in normal fibroblasts [16]. IL-1α might be a potential target for the alleviation of injury on ESCs by suppressing the activation of oxidative stress and the production of inflammatory factors. In *in-vitro* experiments, treatment with IL-1α triggers pro-inflammatory responses in fibroblasts [17]. In epidermal stem cells, IL-1α has also been shown to mediate the inflammatory response and produce the stem cell proliferation factors [18]. Therefore, IL-1α-stimulated ESC cells can be used as an *in-vitro* model.

**Figure 1:**
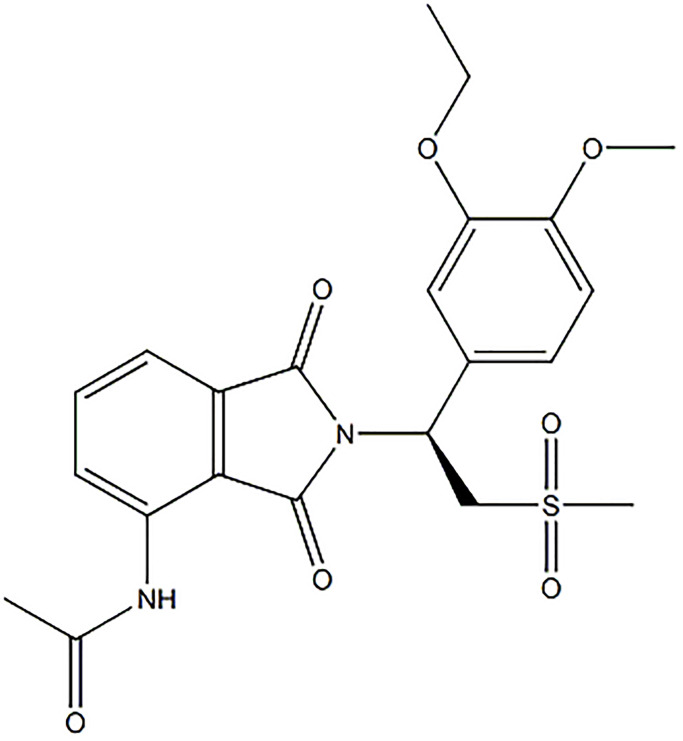
Molecular structure of Apremilast.

Apremilast is a small molecular inhibitor of phosphodiesterase 4 (PDE4) and has been approved by the Food and Drug Administration (FDA) and the European Medicines Agency (EMA) to treat psoriasis vulgaris and joint psoriasis [19]. The molecular structure of Apremilast is shown in Figure 1. PDE4 mediates immunity by degrading the cyclic adenosine monophosphate (cAMP). Apremilast induces the accumulation of cAMP by inhibiting the activity of PDE4 to downregulate the expression level of pro-inflammatory factors and upregulate the expression of anti-inflammatory factors (such as IL-10), which further suppresses the inflammatory reactions [20]. In recent years, Apremilast has been reported to protect skin tissues from external injuries, such as wounds [21]. In the present study, the protective property of Apremilast on IL-1α-induced injury on ESCs, as well as the underlying mechanism, will be investigated to explore the potential therapeutic effects of Apremilast on skin diseases.

## MATERIALS AND METHODS

### Cell isolation and treatments

The protocols for animal experiments used in this study were approved by the Animal Care Committee of The China-Japan Union Hospital of Jilin University. BALB/c mice were purchased from Beijing Vital River Laboratory Animal Technology Co., Ltd. The neonatal mouse dorsal skin was isolated from mice within 1–3 days after birth. Following incubation with 0.3% Dispase II, the ESCs were isolated from the epidermis removed from the animals and cultured in CnT-07 progenitor cell-targeted (PCT) epidermal keratinocyte medium for subsequent experiments. Recombinant mouse IL-1α was purchased from R&D Systems (400-ML), PDE inhibitor Apremilast was from Sigma-Aldrich (SML1099). Cells were stimulated with 5 ng/mL IL-1α in the presence or absence of 1.5 or 3 μM Apremilast for 6 hours. The concentrations of IL-1α and Apremilast were adopted based on the studies previously described [22, 23].

### Real-time PCR analysis

The Trizol Reagent was used to isolate the total RNA from the treated ESCs and cDNA was transformed from the total RNA with a revert aid first-strand cDNA synthesis kit (Fermentas, Vancouver, Canada), followed by real-time PCR analysis with SYBR-Green PCR kit (Invitrogen, California, USA) incubated with cDNA and gene-specific primers. The relative expression levels of related genes were determined using the 2^−ΔΔCt^ method and GAPDH was used as a negative control in the cells. The following primers were used in this study: TNF-α (F: 5′-CCAGACCCTCACACTCAGATC-3′, R: 5′-CACTTGGTGGTTTGCTACGAC-3′); IL-8 (F: 5′-ATGACTTCCAAGCTGGCCGTGGCT-3′, R: 5′-TCTCAGCCCTCTTCAAAAACTTCTC-3′); IL-12 (F: 5′-ATGACTTCCAAGCTGGCCGTGGCT-3′, 5′-AACTTGAGGGAGAAGTAGGAATGG-3′); MMP-2 (F: 5′-AAGGATGGACTCCTGGCACATGCCTTT-3′, R: 5′-ACCTGTGGGCTTGTCACGTGGTGT-3′); MMP-9 (F: 5′-AAGGACGGCCTTCTGGCACACGCCTTT-3′, R: 5′-GTGGTATAGTGGGACACATAGTGG-3′); IL-1R1 (F: 5′-TTTAGCTCACCCATGGCTTCA-3′, R: 5′-GCATCTTGCAGGGTCTTTTCC-3′); Myd88 (F: 5′-CCACCTGTAAAGGCTTCTCG-3′, R: 5′-CTAGAGCTGCTGGCCTTGTT-3′); TRAF6 (F: 5′-ATTTCATTGTCAACTGGGCA-3′, R: 5′-TGAGTGTCCCATCTGCTTGA-3′);

Integrin β1 (F: 5′-ACACCGACCCGAGACCCT-3′, R: 5′-CAGGAAACCAGTTGCAAATTC-3′); Krt19 (F: 5′-GCACTACAGCCACTACTACACGA-3′, R: 5′-CTCATGCGCAGAGCCTGTT-3′), GAPDH (F: 5′-AAGAGGGATGCTGCCCTTAC-3′, 5′-CCATTTTGTCTACGGGACGA-3′).

### Western blot analysis

The proteins extracted from the digested cells were separated using sodium dodecyl sulfate-polyacrylamide gel electrophoresis (SDS-PAGE) [24], followed by being transferred to the PVDF membrane (Thermo Fisher, Massachusetts, USA). The blots were then blocked with 5% BSA to remove the non-specific binding proteins for 1 hour at room temperature. Subsequently, the membranes were incubated with primary antibodies against IL-1R1 (1: 1,000, Abcam, Massachusetts, USA), Myd88 (1: 1,000, Abcam, Massachusetts, USA), TRAF6 (1: 1,000, Abcam, Massachusetts, USA), p-NF-κB p65 (1: 1,000, Abcam, Massachusetts, USA) and β-actin (1: 1,000, Abcam, Massachusetts, USA) at 4°C overnight, followed by being incubated with the secondary antibodies. The immunoreactive bands were visualized by chemiluminescence using an ECL kit (Beyotime, Shanghai, China) and the specific bands were analyzed using Image J software.

### Mito SOX red assay

To determine mitochondrial ROS levels, ESCs were incubated with a mitochondrial superoxide indicator 5 μM MitoSOX Red (YEASEN, Shanghai, China) for 10 minutes at 37°C. The cells were live imaged immediately after incubation with a fresh, complete culture medium for 60 minutes with the Olympus Laser Scanning Confocal Microscope (Olympus, Tokyo, Japan). Quantification of Mito SOX Red staining was performed using the software Image J. Firstly, the regions of interest (ROI) were defined. Secondly, the integrated density value (IDV) of target cells was determined. Thirdly, we counted the average numbers of cells (n) presented in the ROI. Average levels of mitochondrial ROS = IDV/n.

### ELISA assay

ELISA assay was used to detect the concentrations of TNF-α, IL-8, IL-12, MMP-2, and MMP-9 in the supernatant of treated ESCs that were incubated with 5% BSA to remove the non-specific binding proteins for 1 hour at room temperature, followed by incubation with the primary antibodies for 1 hour. Subsequently, the samples were incubated with streptavidin-horseradish peroxidase (HRP)-conjugated secondary antibodies for 20 minutes at room temperature, followed by being read at 450 nm with a microplate spectrophotometer (Thermo Fisher, Massachusetts, USA).

### Luciferase reporter assay

The cells were seeded on the 24-well plates at a density of 5 × 10^4^ cells/well and transfected with the pNL3.2-NF-κB-RE reporter plasmids using TransFast transfection reagent (Promega, Madison, USA). The luciferase reporter assay was performed as described previously [20]. Briefly, transfected ESCs were allowed to grow for 24 hours after the transfection, followed by stimulation with 5 ng/mL IL-1α in the presence or absence of 1.5 or 3 μM Apremilast for 6 hours. The cell extracts were prepared with reporter lysis buffer and luciferase activity was measured with a dual luciferase assay buffer (Promega). The transfection efficiencies were normalized to an independent control (Renilla luciferase vector). The results were presented as the fold increase with untreated cells as control.

### Caspase activity assay

For the determination of caspase activity, the Caspase 3/7 assay kit was used. ESCs were seeded at a density of 1 × 10^5^ cells/mL in a white-walled 96-well plate. At the indicated time points, cells were assayed using the respective kits according to the manufacturer’s instructions. Caspase 3/7 assay buffer was added and incubated at 22°C for 30 minutes. The luminescence was then recorded.

### Statistical analysis

GraphPad Prism 7.0 (GraphPad Software, USA) was used to perform statistical analysis. Data are presented as Mean  ±  SD. Results were statistically analyzed with Student’s *t*-test for group comparisons and using one-way ANOVA with Tukey’s post hoc test for multiple group comparisons. Ns indicates no significant difference. Data with *P* values < 0.05 were considered significant.

## RESULTS

### Apremilast suppressed IL-1α-induced expression of pro-inflammatory cytokines in mouse ESCs

**Figure 2:**
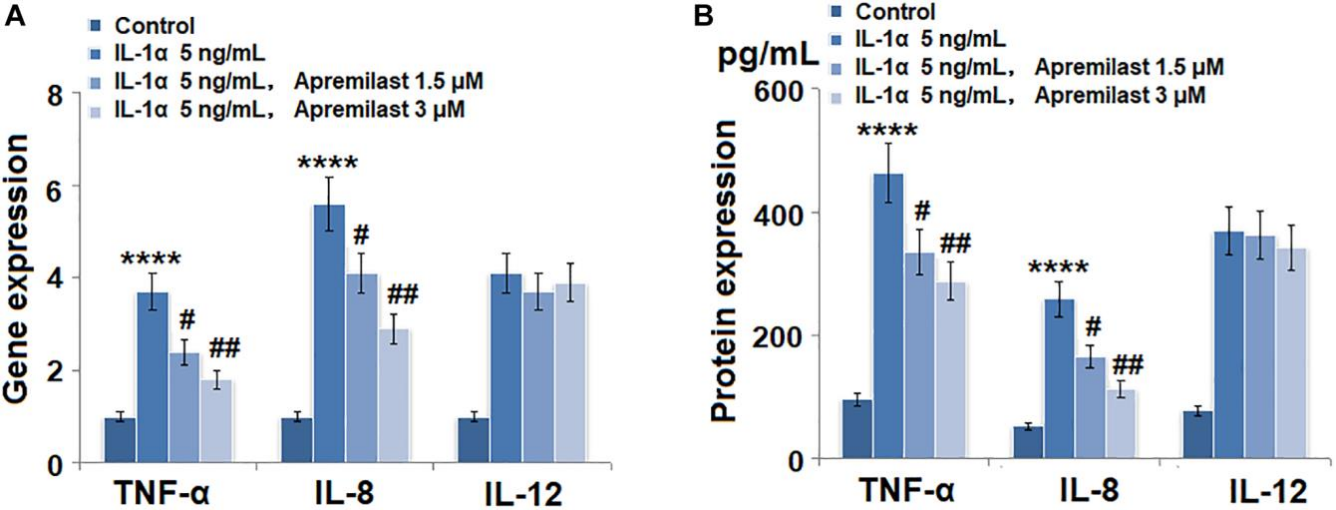
**Apremilast suppressed IL-1α-induced expression of pro-inflammatory cytokines in mouse epidermal stem cells (ESCs).** Cells were stimulated with 5 ng/mL IL-1α in the presence or absence of 1.5 or 3 μM Apremilast for 12 hours. (**A**). mRNA of TNF-α, IL-8, and IL-12; (**B**). Secretions of TNF-α, IL-8, IL-12 (^****^P < 0.0005 vs. vehicle group; ^#^, ^##^, *P* < 0.05, 0.01 vs. IL-1α treatment group, *N* = 5–6).

To evaluate the effects of Apremilast on the inflammation in the ESCs induced by IL-1α, the cells were stimulated with 5 ng/mL IL-1α in the presence or absence of 1.5 or 3 μM Apremilast for 12 hours and the concentrations of inflammatory factors were detected. As shown in Figure 2A, the expression levels of TNF-α, IL-8 and IL-12 were significantly elevated by stimulation with IL-1α. Importantly, treatment with Apremilast significantly inhibited the elevated expressions of TNF-α and IL-8, but not IL-12. ELISA assay was used to detect the concentrations of the inflammatory factors. As shown in Figure 2B, the concentrations of TNF-α in the control, IL-1α, 1.5 μM Apremilast and 3 μM Apremilast groups were 96.2, 462.4, 335.4 and 288.1 pg/mL, respectively. Approximately 52.7, 259.1, 165.8, and 113.3 pg/mL IL-8 were determined in the ESCs incubated with blank medium, IL-1α, IL-1α in the presence of 1.5 μM Apremilast and IL-1α in the presence of 3 μM Apremilast, respectively. In addition, the concentrations of IL-12 in the control, IL-1α, 1.5 μM Apremilast, and 3 μM Apremilast groups were 78.5, 369.6, 361.7, and 342.7 pg/mL, respectively.

### The expressions of MMP-2 and MMP-9 induced by IL-1α were prevented by apremilast in mouse ESCs

**Figure 3:**
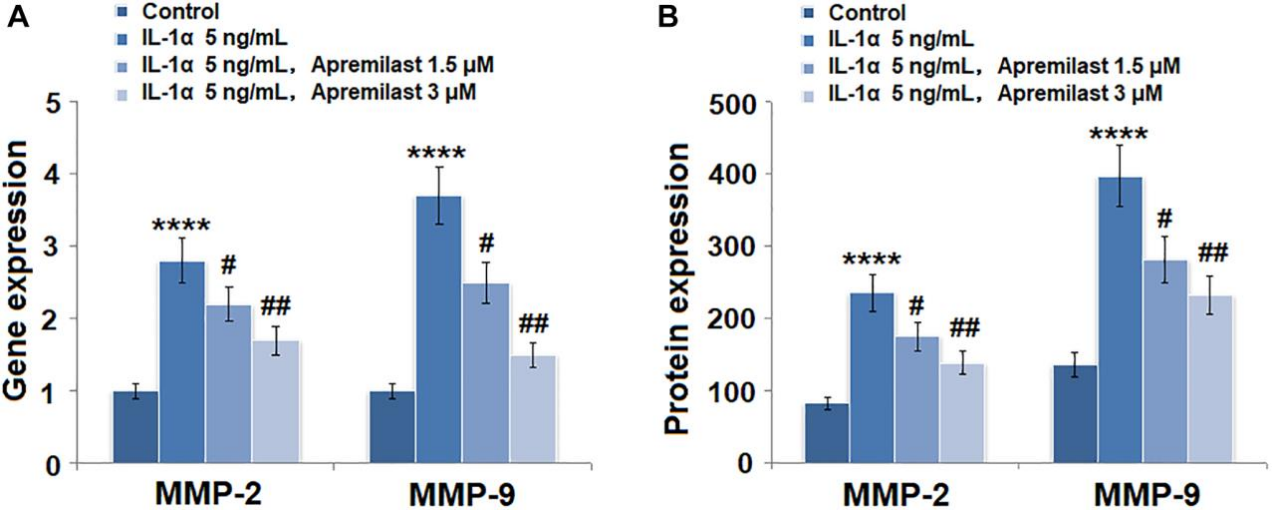
**Apremilast prevented IL-1α-induced expression of MMP-2 and MMP-9 in ESCs.** Cells were stimulated with 5 ng/mL IL-1α in the presence or absence of 1.5 or 3 μM Apremilast for 12 hours. (**A**). mRNA of MMP-2 and MMP-9; (**B**). Protein of MMP-2 and of MMP-9 (^****^P < 0.0005 vs. vehicle group; ^#^, ^##^, *P* < 0.05, 0.01 vs. IL-1α treatment group, *N* = 5–6).

As shown in Figure 3A, we found that the elevated expressions of MMP-2 and MMP-9 induced by stimulation with 5 ng/mL IL-1α were significantly inhibited by treatment with Apremilast in a dose-dependent manner. The concentrations of MMP-2 in the control, IL-1α, 1.5 μM Apremilast, and 3 μM Apremilast groups were 83.5, 235.6, 176.2, and 138.9 pg/mL, respectively. Approximately 136.7, 397.1, 281.5, and 233.5 pg/mL MMP-9 were detected in the ESCs treated with blank medium, IL-1α, IL-1α in the presence of 1.5 μM Apremilast and IL-1α in the presence of 3 μM Apremilast, respectively (Figure 3B).

### Apremilast alleviated IL-1α-induced oxidative stress and expression of IL-1R1 in ESCs

**Figure 4:**
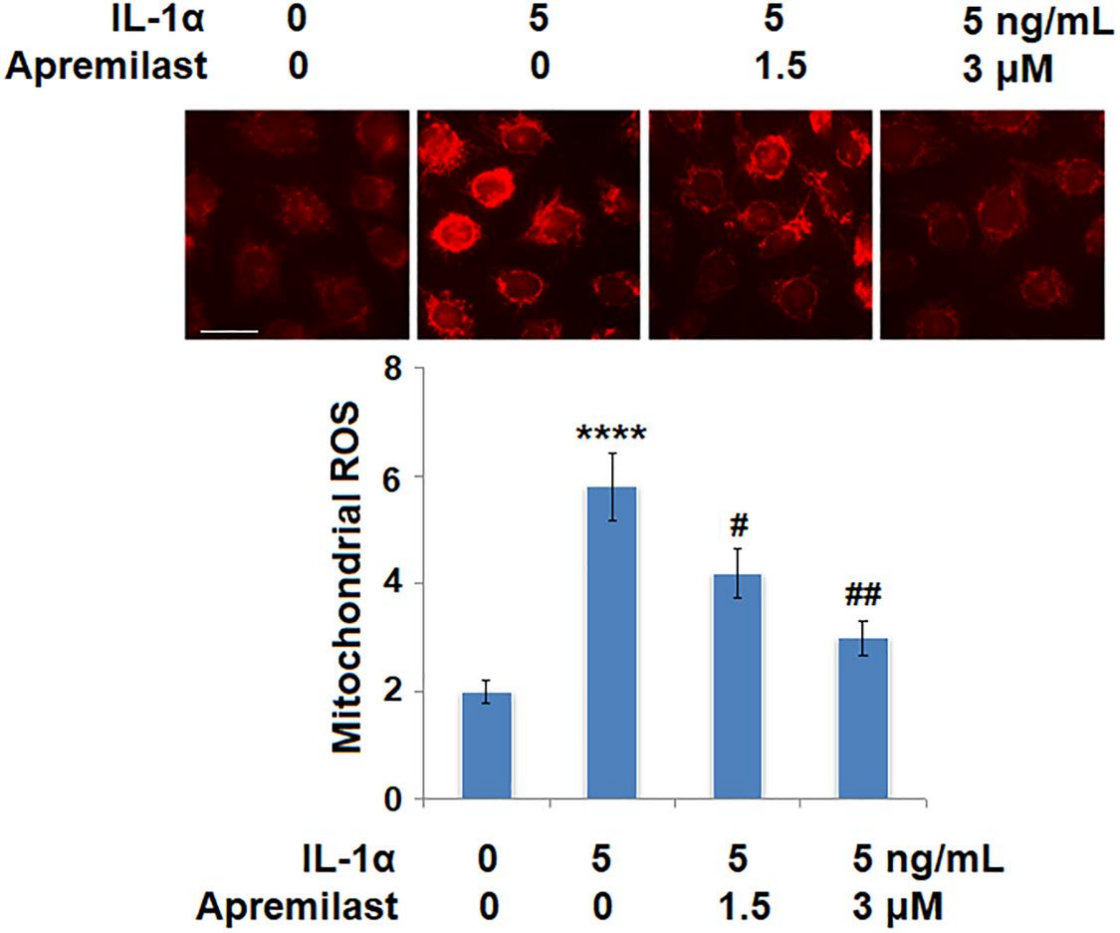
**Apremilast alleviated IL-1α-induced oxidative stress in ESCs.** Cells were stimulated with 5 ng/mL IL-1α in the presence or absence of 1.5 or 3 μM Apremilast for 12 hours. Mitochondrial ROS (^****^P < 0.0005 vs. vehicle group; ^#^, ^##^, *P* < 0.05, 0.01 vs. IL-1α treatment group, *N* = 6) was measured using MitoSOX Red. Scale bar, 100 μm.

**Figure 5:**
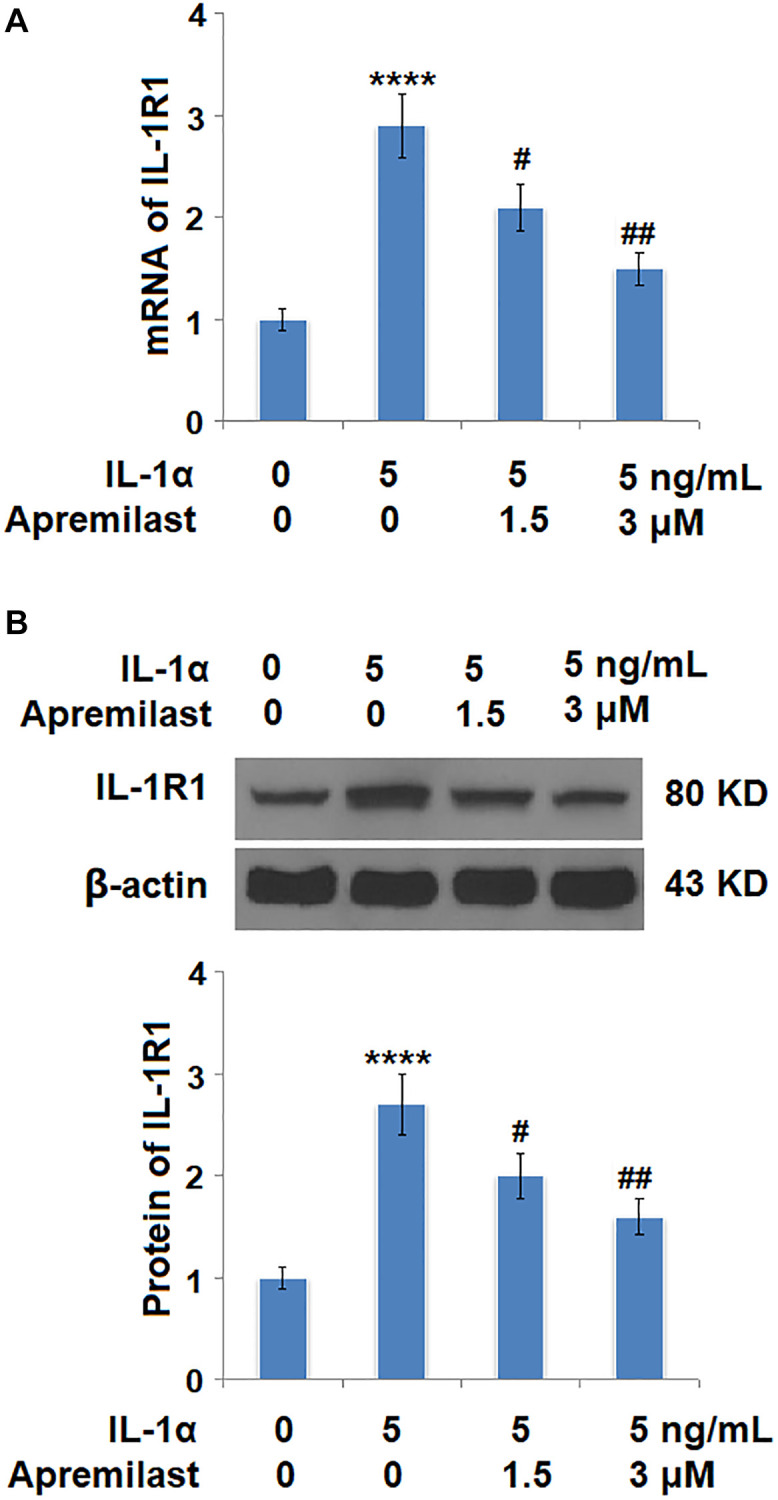
**Apremilast reduced IL-1α-induced expression of IL-1R1 in ESCs.** Cells were stimulated with 5 ng/mL IL-1α in the presence or absence of 1.5 or 3 μM Apremilast for 12 hours. (**A**). mRNA of IL-1R1; (**B**). Protein of IL-1R1 (^****^P < 0.0005 vs. vehicle group; ^#^, ^##^, *P* < 0.05, 0.01 vs. IL-1α treatment group, *N* = 5–6).

Oxidative stress was evaluated by measuring mitochondrial ROS. As shown in Figure 4, the mitochondrial ROS levels were significantly increased by stimulation with IL-1α but greatly suppressed by treatment with Apremilast. In addition, the upregulated expression of IL-1R, the main binding receptor of IL-1α, induced by stimulation with IL-1α, was significantly downregulated by the introduction of Apremilast at both the mRNA and protein levels (Figure 5A and 5B).

### Apremilast mitigated IL-1α-induced apoptosis in ESCs

**Figure 6:**
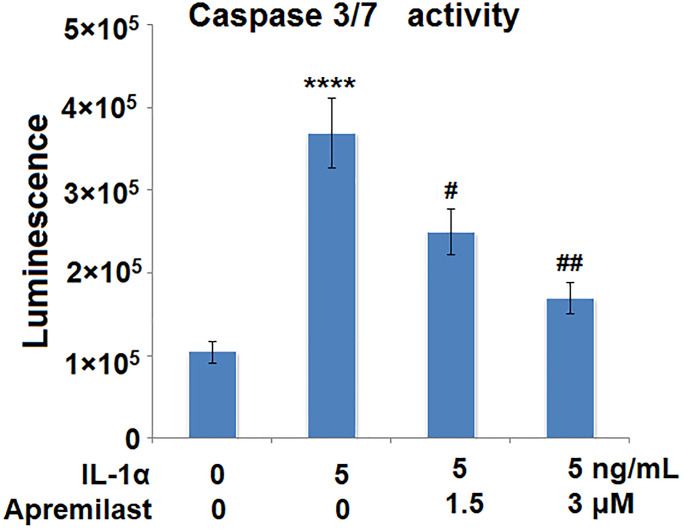
**Apremilast mitigated IL-1α-induced apoptosis in ESCs.** Cells were stimulated with 5 ng/mL IL-1α in the presence or absence of 1.5 or 3 μM Apremilast for 12 h. The activity of caspase 3/7 as measured using a commercial kit (^****^P < 0.0005 vs. vehicle group; ^#^, ^##^, *P* < 0.05, 0.01 vs. IL-1α treatment group, *N* = 5–6).

The activity of caspase 3/7 has been considered as a marker for apoptosis. We used it to investigate the effects of Apremilast in IL-1α-induced apoptosis. Results in Figure 6 demonstrate that IL-1α treatment significantly increased the activity of Caspase 3/7, which was significantly reduced by Apremilast in a dose-dependent manner. These findings suggest that Apremilast mitigated IL-1α-induced apoptosis in ESCs.

### Apremilast suppressed the Myd88/TRAF6/NF-κB p65 signaling induced by IL-1α

**Figure 7:**
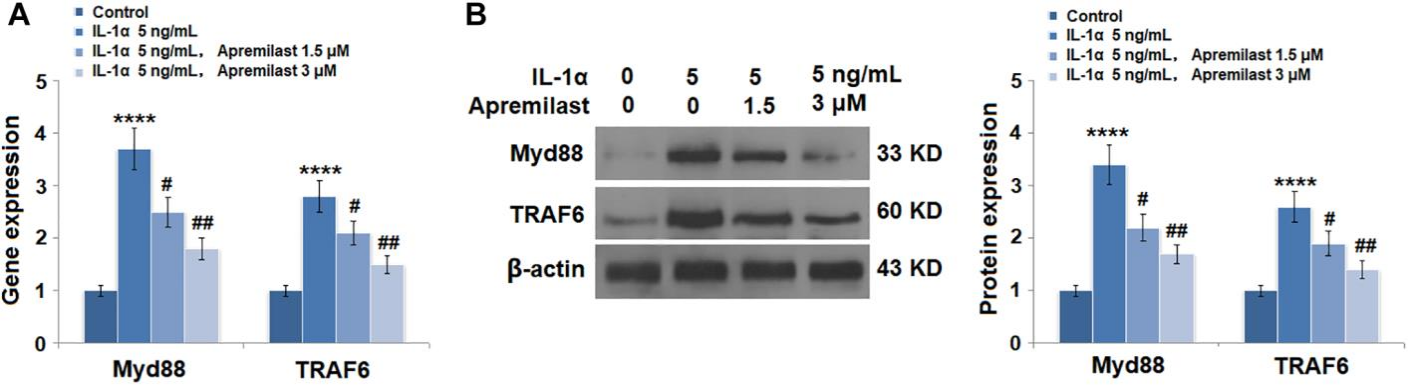
**Apremilast reduced IL-1α-induced expressions of Myd88 and TRAF6 in ESCs.** Cells were stimulated with 5 ng/mL IL-1α in the presence or absence of 1.5 or 3 μM Apremilast for 12 hours. (**A**). mRNA of Myd88 and TRAF6; (**B**). Protein of Myd88 and TRAF6 (^****^P < 0.0005 vs. vehicle group; ^#^, ^##^, *P* < 0.05, 0.01 vs. IL-1α treatment group, *N* = 5–6).

**Figure 8:**
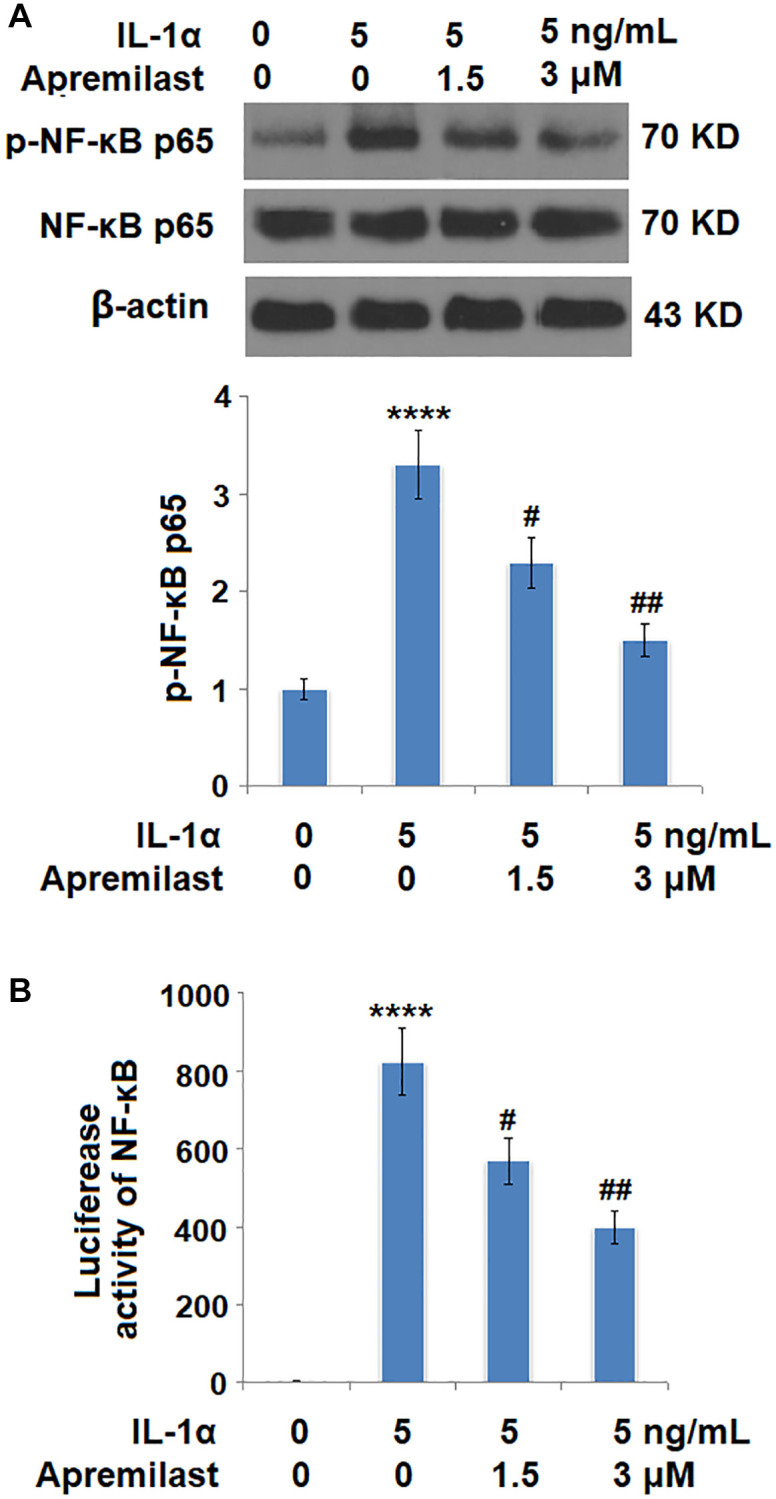
**Apremilast inhibited IL-1α-induced activation of NF-kB in ESCs.** Cells were stimulated with 5 ng/mL IL-1α in the presence or absence of 1.5 or 3 μM Apremilast for 6 hours. (**A**). Levels of p-NF-κB p65; (**B**). Luciferase activity of NF-κB (^****^P < 0.0005 vs. vehicle group; ^#^, ^##^, *P* < 0.05, 0.01 vs. IL-1α treatment group, *N* = 5–6).

To explore the potential mechanism underlying the inhibitory effect of Apremilast against IL-1α-induced excessive inflammation and oxidative stress, the expression levels of Myd88 and TRAF6, as well as the activation state of NF-κB p65, were detected after the cells were stimulated with 5 ng/mL IL-1α in the presence or absence of 1.5 or 3 μM Apremilast for 6 hours. As shown in Figure 7, the elevated expression levels of Myd88 and TRAF6 induced by treatment with IL-1α were greatly inhibited by the introduction of Apremilast in a dose-dependent manner. In addition, the expression level of p-NF-κB p65 was significantly promoted by IL-1α but suppressed by treatment with Apremilast (Figure 8A) in a dose-dependent manner. Importantly, as shown in Figure 8B, the enhanced luciferase activity of NF-κB induced by stimulation with IL-1α was greatly reduced by the introduction of Apremilast in a dose-dependent manner.

### Apremilast protected ESCs against IL-1α-induced impairment in capacities of ESCs

**Figure 9:**
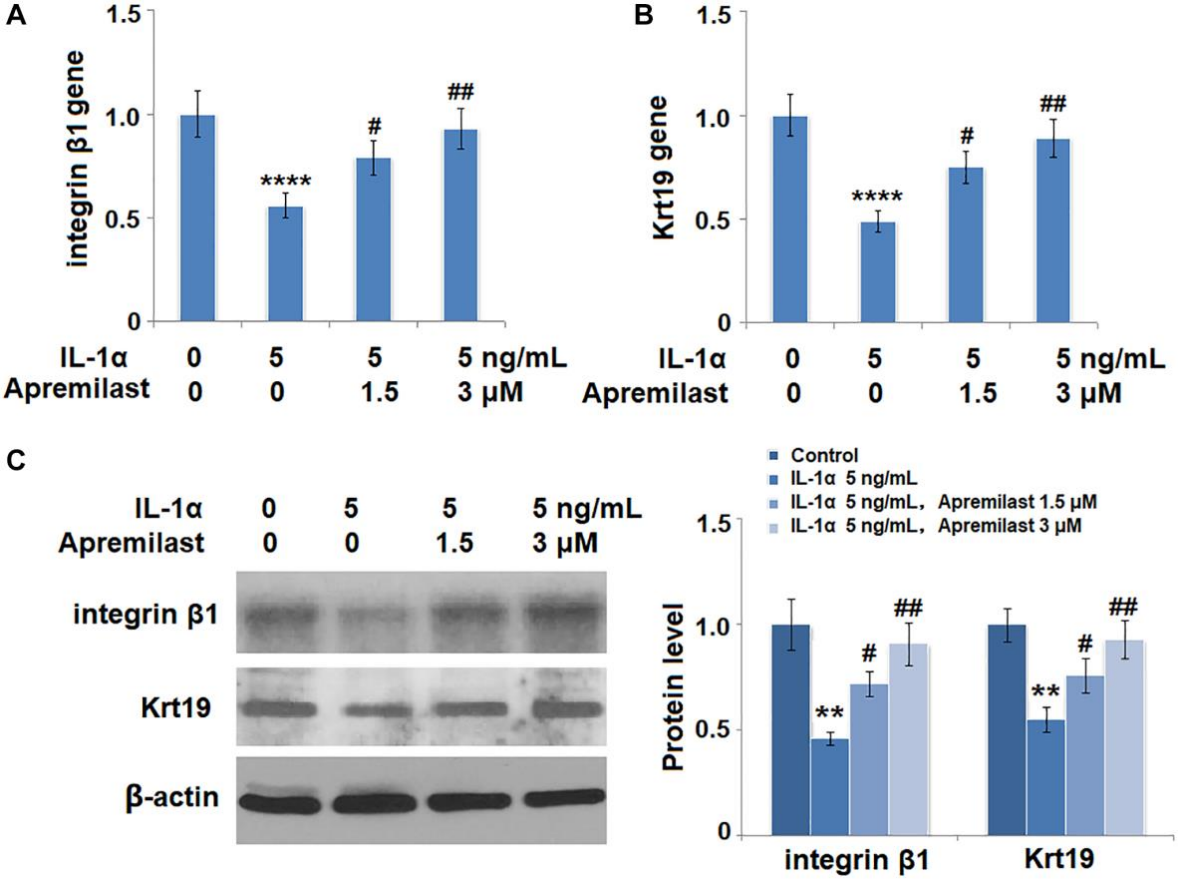
**Apremilast protects ESCs against IL-1α-induced impairment in capacities of ESCs.** Cells were stimulated with 5 ng/mL IL-1α in the presence or absence of 1.5 or 3 μM Apremilast for 12 hours. (**A**) mRNA of integrin β1; (**B**). mRNA of Krt19; (**C**). Protein levels of integrin β1 and Krt19 (^**^, ^****^, *P* < 0.01, 0.0005 vs. vehicle group; ^#^, ^##^, *P* < 0.05, 0.01 vs. IL-1α treatment group, *N* = 5).

To evaluate the impact of Apremilast on the biological function of ESCs, the gene expressions of integrin β1 and Krt19 were determined in the ESCs following stimulation with 5 ng/mL IL-1α in the presence or absence of 1.5 or 3 μM Apremilast for 12 hours. As shown in Figure 9A and 9B, the expression levels of integrin β1 and Krt19 at the mRNA level were significantly decreased by stimulation with IL-1α but greatly elevated by the introduction of Apremilast. Consistently, Apremilast preserved the expressions of integrin β1 and Krt19 at the protein level (Figure 9C), indicating a promising protective property of Apremilast against IL-1α-induced impairment in capacities of ESCs.

## DISCUSSION

A group of cells that are highly expressed with integrin α2β1 or α3β1 are located in the epidermal basal layer. They are characterized by clonal growth, are small in size, have rare organelles, and a low concentration of cellular RNA [25]. These cells are defined as ESCs. As the expression level of integrin β1 in the ESCs is approximately 2-fold higher than that in other cells, integrin β1 has been regarded as the specific biomarker of ESCs [26]. In the process of re-epithelialization of the wound, ESCs differentiate into various layers of epidermal cells to contribute to the repair of injured skin tissues [27]. In an investigation on the distribution of regenerative cells in the wound repairing tissues, Krt19-positive ESCs were observed in the epidermal basal layer of repairing tissues. In addition, in the layer between basal cells and keratinocytes, numerous Krt19-positive ESCs were observed. However, in the normal skin tissues, Krt19-positive ESCs were only located in the epidermal basal layer and the cutaneous appendages, indicating that ESCs might be involved in the process of wound repairing [28]. However, external factors, such as UV and inflammation infiltration, bring significant injury to ESCs, therefore reducing their tissue repairing ability [29, 30]. A series of pathological processes can be induced when the ESCs are injured, including oxidative stress and inflammation [31]. Oxidative stress is characterized by excessively produced ROS. Under a physiological state, a balance between the production and elimination of ROS is maintained by the anti-oxidative and oxidative systems. When the excessively produced ROS cannot be excluded by the anti-oxidative system, cellular or tissue injuries are induced, and this is widely regarded as oxidative stress [32]. In the present study, IL-1α was used as an inflammatory stimulator to induce an *in-vitro* injury model on ESCs, it was verified by excessive excretion of inflammatory factors, an elevated level of oxidative stress, and decreased expression of ESCs biomarkers. Recent investigations indicate that two subtypes of pro-inflammatory cytokines are produced in ESCs during wound healing. One subtype is induced in the early response to antigens, such as TNF-α and IL-8. Another type is mainly involved in the tissue repair process, like IL-12 and IL-10. The balanced regulation of these pro- inflammatory cytokines is critical to epidermal cell function and wound healing [33]. In this study, we found that Apremilast suppressed IL-1α-induced expressions of TNF-α and IL-8. The blunt effect of Apremilast on IL-12 could be related to its function in late stage tissue repair. Through treatment with Apremilast, we found that the expressions of integrin β1 and Krt19 were significantly upregulated and the levels of mitochondrial ROS were greatly suppressed. The key proteins of the down-stream of oxidative stress MMP-2 and MMP-9 were also significantly downregulated. These data indicate that Apremilast protects ESCs against IL-1α-induced impairment in capacities by suppressing the level of oxidative stress. Based on the prospective results in the present study, the molecular mechanism underlying the regulatory effect of Apremilast on oxidative stress will be further explored in our future work.

Severe inflammation was another important pathological process induced by IL-1α in ESCs. The TLR4/Myd88/TRAF6 signaling pathway is a classic inflammatory signaling pathway, reported to exert an important role in the development and process of fibrosis. By regulating the NF-κB signaling pathway, the TLR4/MyD88/TRAF6 signal triggers the excessive production of inflammatory factors to induce and aggravate the pathological state of fibrosis [34, 35]. As an important pathway involved in the regulation of inflammatory factors secretion and production, the NF-κB signaling pathway was not activated under a normal physiological state. IκB, an innate inhibitor for NF-κB, blocks the gene mapping sequence of NF-κB by binding with NF-κB and inhibits the biological function of NF-κB. However, the phosphorylation of IκB is triggered when the cells are stimulated by external or internal factors, such as inflammatory mediators, viral infections, and oxidative stress. In this way, NF-κB is released from the inhibitory state, and transferred to the cell nucleus, and bound with enhancers of target proteins. As a result, transcription is enhanced [36, 37]. A series of studies show that Apremilast could ameliorate drug-induced cardiovascular toxicity by the inhibition of the ERK and JNK kinases, and NF-κB/inflammatory pathways [38–39]. Particularly, Apremilast acts to inhibit the NF-κB pathway by upregulating IL10 and downregulating TNF-α [40]. PDE4 inhibition causes intracellular accumulation of cAMP-responsive element-binding protein (CREB/ATF-1) family of transcription factors. Apremilast is also reported to modulate inflammatory response by enhancement of cAMP-responsive element (CRE)-driven gene transcription and inhibition of NF-κB-driven gene transcription [41]. Our data show that treatment with Apremilast significantly reduced the production of inflammatory factors and activation of the Myd88/TRAF6 signaling pathway by IL-1α. The levels of p-NF-κB p65 were significantly suppressed, indicating that Apremilast might inhibit the severe inflammation in the ESCs induced by IL-1α through regulating the Myd88/TRAF6/NF-κB signaling pathway. However, the direct target of Apremilast still remains to be identified. Therefore, the protective effect of Apremilast could be mediated by the NF-κB/TLR pathway in ESCs. In our future work, TLR4 will be further investigated to claim whether it is the direct or indirect target of Apremilast that is responsible for the biological functions achieved in the present study.

Taken together, we conclude that Apremilast might ameliorate IL-1α-induced dysfunction in ESCs by alleviating oxidative stress and inflammation.
