# Correction for: Apremilast ameliorates IL-1α-induced dysfunction in epidermal stem cells

**DOI:** 10.18632/aging.205318

**Published:** 2024-03-31

**Authors:** Yuxi Jia, Xiangru Chen, Jing Sun

**Affiliations:** 1 Department of Dermatology, The China-Japan Union Hospital of Jilin University, Changchun, Jilin 130033, China

**Keywords:** Apremilast, ESCs, IL-1α, oxidative stress, inflammation

**This article has been corrected:** The authors found that the Western blot images for p-NF-κB p65 and β-actin in **Figure 8A** and the Krt19 image in **Figure 9C** were incorrect due to mislabeling and misuse of data from a different experiment. The authors have provided uncropped images of the original blots and replaced the incorrect images with images from the original experiments. These corrections have no impact on the experimental outcome or conclusions. The authors would like to apologize for any inconvenience caused.

The corrected **Figures 8** and **9** are shown below.

**Figure 8:**
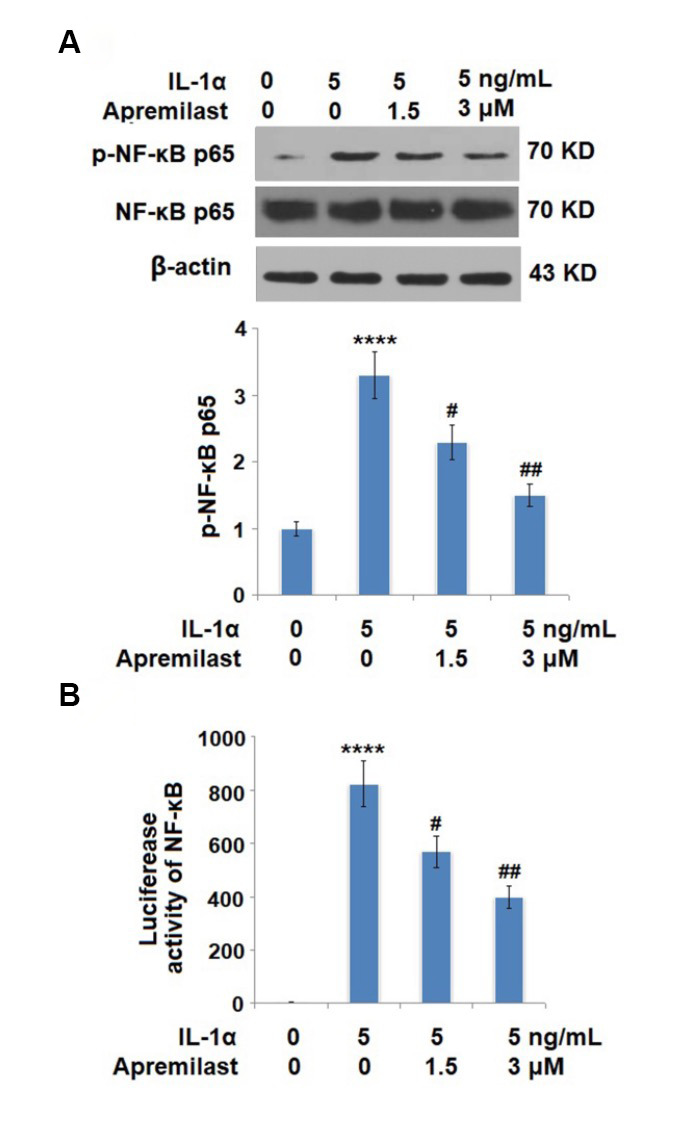
**Apremilast inhibited IL-1α-induced activation of NF-kB in ESCs.** Cells were stimulated with 5 ng/mL IL-1α in the presence or absence of 1.5 or 3 μM Apremilast for 6 hours. (**A**). Levels of p-NF-κB p65; (**B**). Luciferase activity of NF-κB (****P < 0.0005 vs. vehicle group; ^#^, ^##^, *P* < 0.05, 0.01 vs. IL-1α treatment group, *N* = 5–6).

**Figure 9:**
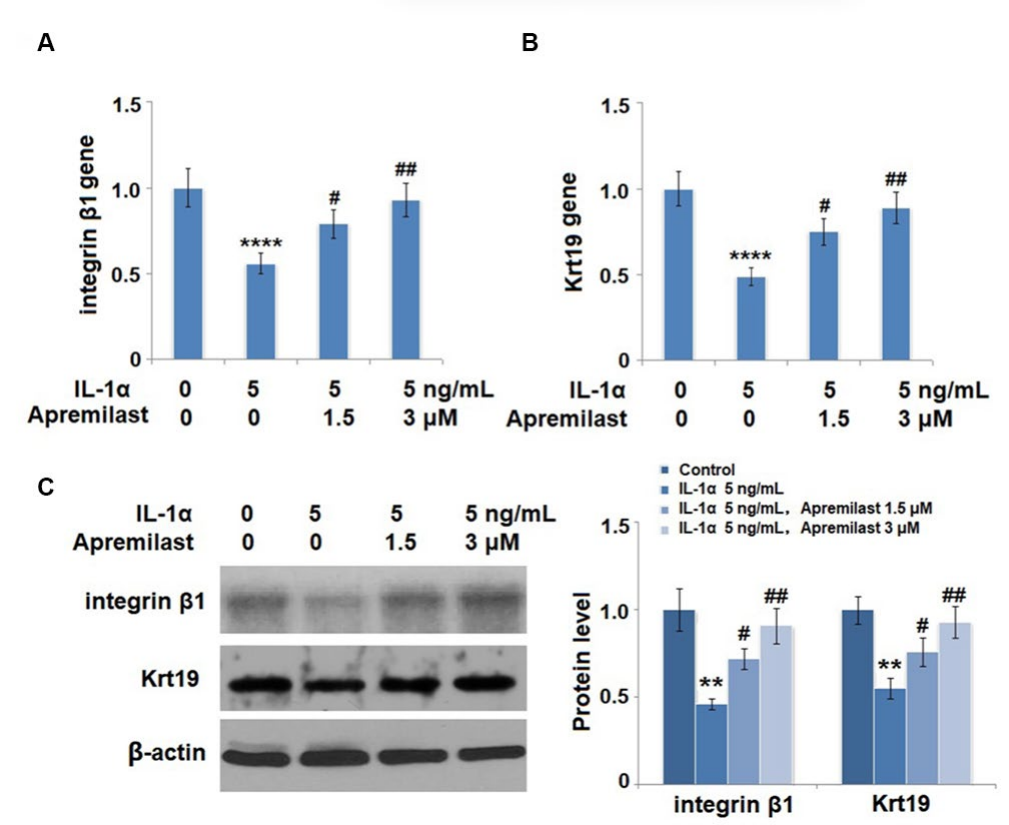
**Apremilast protects ESCs against IL-1α-induced impairment in capacities of ESCs. **Cells were stimulated with 5 ng/mL IL-1α in the presence or absence of 1.5 or 3 μM Apremilast for 12 hours. (**A**) mRNA of integrin β1; (**B**). mRNA of Krt19; (**C**). Protein levels of integrin β1 and Krt19 (**, ****, *P* < 0.01, 0.0005 vs. vehicle group; ^#^, ^##^, *P* < 0.05, 0.01 vs. IL-1α treatment group, *N* = 5).

